# Gender-dependent impact of COVID-19 lockdown on metabolic and psychological aspects

**DOI:** 10.1007/s11739-022-03173-9

**Published:** 2023-01-27

**Authors:** Leonilde Bonfrate, Agostino Di Ciaula, Mohamad Khalil, Ilaria Farella, Roberta Chirico, Gemma Vilahur, Piero Portincasa

**Affiliations:** 1 Department of Biomedical Sciences and Human Oncology, Clinica Medica A. Murri, University of Bari Aldo Moro, 70124 Bari, Italy; 2 Research Institute, Hospital de la Santa Creu i Sant Pau, IIB-Sant Pau, Barcelona, Spain; 3 CiberCV, Instituto Salud Carlos III, Barcelona, Spain

**Keywords:** Gender, COVID-19, Metabolic disease, Psychological aspects, Mediterranean diet

## Abstract

The first COVID-19 lockdown resulted in enforced quarantine of heavily affected areas with social isolation and related measures by several governments to slow the spread of the disease. The general population experienced several mental and lifestyle changes. Herein, we aimed to evaluate the metabolic and psychological effects induced by lifestyle changes during COVID-19 self-isolation among an Apulian overweight/obese cohort with metabolic disturbances. The study assessed anthropometric data (weight, abdominal circumferences), dietary habits (adherence to the Mediterranean diet, junk food score), lifestyle habits (i.e., smoking, and physical activity), levels of stress and anxiety, and depression. Subjects underwent bioumoral exams before and after self-isolation to monitor glycemic and lipid profiles. A total of 245 subjects (*M*:*F* = 118:127) have been included in the study. After lockdown, the number of obese subjects significantly increased in both sexes, and was higher in females than in males (*P* < 0.0001). Glycemic and lipid profiles worsened, with higher levels of insulinemia, lower levels of HDL cholesterol, and higher levels of triglycerides in females than in males. Adherence to the Mediterranean diet and consumption of junk foods were altered in both groups, especially in females. Psychological aspects were significantly higher in females than in males. Finally, work activities and familial status strongly affected the metabolic and psychological profile. In conclusion, COVID-19 self-isolation induced changes in lifestyle and dietary habits with psychological distress and detrimental effects on metabolic patterns, which were more pronounced in female gender.

**Supplementary Information:**

The online version contains supplementary material available at 10.1007/s11739-022-03173-9.

## Introduction

The new coronavirus infection caused by severe acute respiratory syndrome coronavirus 2 (SARS-CoV-2) rapidly involved Europe and spread throughout the world. The COVID-19 epidemic was defined a pandemic, as declared by the World Health Organization (WHO) statement on 11 March 2020 [1] (accessed March 16, 2020). Italy was one of the earliest and most deeply affected countries with 80,589 confirmed cases of COVID-19 and a highest mortality rate (8215) by March 26 [2]. During the State of Emergency, various government authorities adopted the full lockdown aiming to limit the spread of the virus and the collapse of health care systems. These measures led to changes in lifestyle behaviors. All opportunities of socializing were blocked resulting in isolation and changes in working activities [3]. The lockdown worsened the sedentary habits in adult population [4], which is associated with an increased risk of cardio-metabolic disorders [5, 6]. The effects COVID-19 lockdown were mainly the decreased adherence to a healthy diet, the increased consumption of sugary “*comfort foods*” as a response to negative emotions, variation in sleeping, smoking, and work habits, which resulted in detrimental metabolic effects and increased cardio-metabolic risk [3, 7, 8]. The unhealthy habits during pandemic lockdowns have exposed people to weight gain and increased visceral fat, which causes a chronic inflammatory state and an increased risk of more serious complications and clinical outcome in patients with COVID-19 [9–13].

The "metabolic urgency" of COVID-19 lockdown has been recognized by several national and international organizations which have published recommendations related to food and nutrition during the period of lockdown [14]. Moreover, during the state of emergency, the priority was for COVID-19 patients and the outpatient hospital visits for follow-up or prevention of non-communicable diseases were limited to virtual consultations [15–18]. Difficulties in the access to health care services and the lockdown itself increased the risk for anxiety and stress [19] as well as an increased risk for psychiatric illness associated with COVID-19 [20–23]. Some studies showed that women especially experimented more depression and anxiety during COVID-19 lockdown [24]. A gender pre-existing trend [25, 26] was probably exacerbate during a stressful time as the pandemic [27]. However, the gender-dependent impact of COVID-19 lockdown on metabolic and psychological aspects still remains incompletely explored. The aim of this observational study was therefore to investigate the impact of COVID-19 self-isolation on metabolic and psychological profiles, looking at gender differences among a cohort of overweight/obese subjects.

## Methods

### Study design

**Fig. 1:**
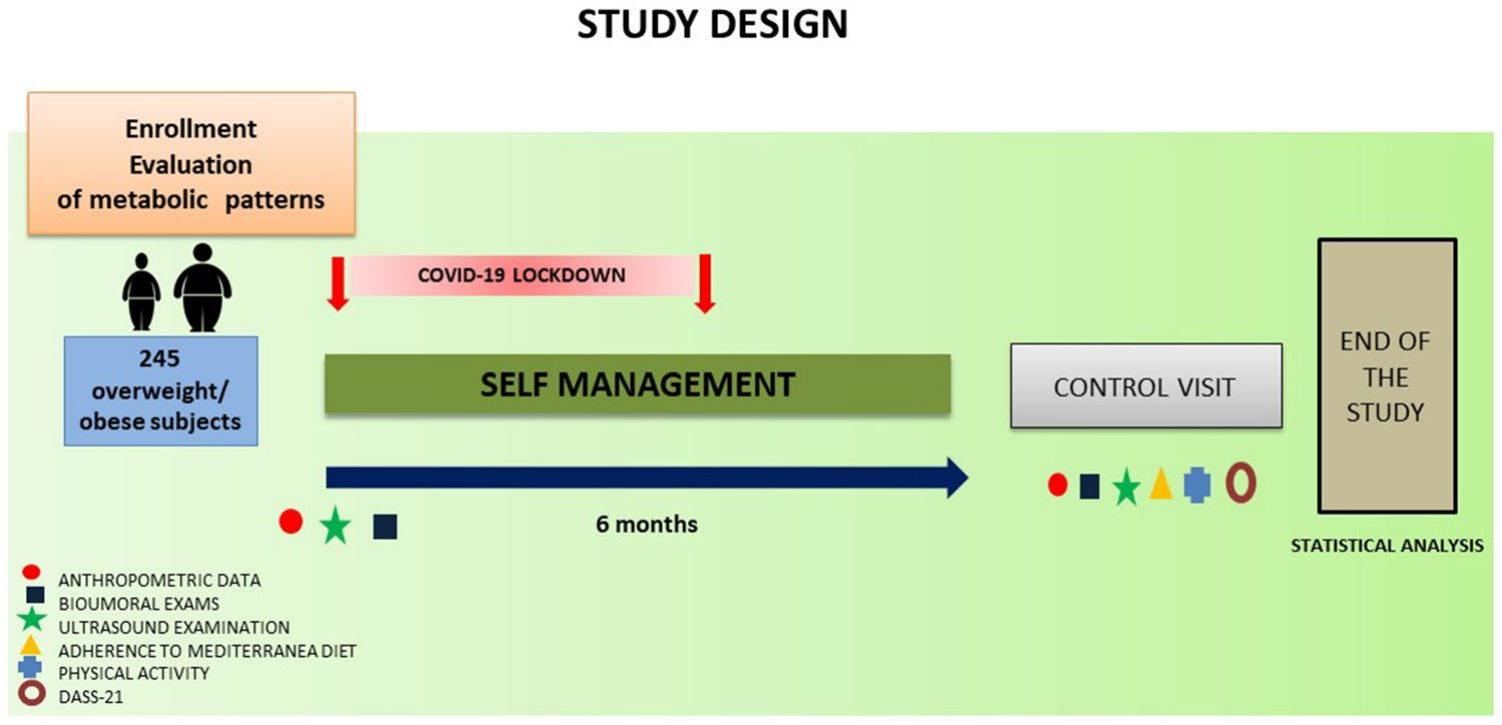
Study design (see text for details)

The general characteristics of the study are depicted in Fig. 1S. The study started in January 2020, and a total of 280 overweight/obese outpatient adult subjects (146 females) were screened for the clinical assessment of metabolic abnormalities at the Division of Internal Medicine at the University of Bari. The average duration of the study was 4.8 ± 1.2 months from the enrollment.

Subjects were enrolled in the presence of a Body Mass Index (BMI) > 24.9 kg/mt^2^, associated with one or more of the following clinical conditions: type 2 diabetes, blood hypertension, metabolic-associated fatty liver disease (MAFLD), increased waist circumference, and altered serum lipid profile. Exclusion criteria included diagnosis of infectious diseases (including SARS-CoV-2), inflammatory bowel disorders, abdominal surgery within the previous six months, drug or alcohol abuse, mental illness (including eating disorders), concomitant immunological, hematological or neoplastic diseases, hepatic failure (i.e., Child–Pugh class C), and severe heart failure (NYHA class III–IV). A total of 18 enrolled subjects were excluded from the study due to COVID-19 hospitalization, and 17 patients dropped out. Thus, the final cohort included 245 subjects. At entry, all subjects underwent a general and clinical assessment that included:

o Socio-demographic information: gender, age, nationality, occupational status, place of residence and living situation at home
o BMI calculation and subsequent body size classification according to the National Institutes of Health and WHO criteria: underweight (BMI < 18.5 kg/m^2^); normal weight (BMI ≥ 18.5–24.9 kg/m^2^); overweight (BMI ≥ 25.0–29.9 kg/m^2^); obese (BMI ≥ 30 kg/m^2^).
o Measurement of waist circumference. Waist circumference was measured on a horizontal plane at the equidistant point between the lowest floating rib and the upper border of the iliac crest. A waist circumference ≥ 94 cm for males and ≥ 80 cm for females was considered indicative of abdominal obesity and increased cardio-metabolic risk [28].
o Medical history. The presence of diabetes, hypertension, MAFLD, dyslipidemia was carefully evaluated according to available diagnostic guidelines. When present, ongoing treatments were recorded.

Following the basal evaluation (at entry), all patients underwent a regular follow-up assessment (after the lockdown) (Fig. 1) including:

- BMI calculation and measurement of waist circumference;
- Venous sampling to assess glycemic profile (glycemia, normal value < 100 mg/dl; insulinemia, normal value 2.6–24.9 microUI/ml; and glycated hemoglobin in the case of diabetic patients, normal value < 48 mmol/mol), lipid profile (total cholesterol, normal value < 200 mg/dl; HDL cholesterol, normal value > 40 mg/dl for man and > 50 mg/dl for woman; LDL < 160/130/100/70 mg/dl stratified for cardiovascular risk; and triglycerides, normal value < 150 mg/dl), alanine transaminase (ALT) (normal value 15–37 U/L), aspartate transaminase (AST) (normal value 12–78 U/L), gamma-glutamyl transferase (GGT) (normal value 15–85 U/L)
- Ultrasound abdominal examination to detect the degree of steatosis based on Liver-to-kidney echogenicity [29].
- Adherence to Mediterranean dietary pattern by analyzing nine food categories with a score ranging from 0 point (lowest adherence) to 18 points (highest adherence) [30].
- The alcohol consumption: number daily drinks
- Assessment of physical activity (performing at least 150–300 min of moderate or 75–150 min of vigorous physical activity a week or a combination of these, and muscle strengthening activities at least twice a week) [31] using MEDSTYLE, a custom-designed questionnaire, tested across different ages, and anthropometric groups in health and disease [32].
- Assessment of junk food consumption. Previous evidence documented that home confinement was linked with sedentary lifestyle and with increased consumption of junk food [33] The term “junk food” includes unhealthy food characterized by high calories from sugar and/or fat, and low-nutrient value (i.e., bakery products, beverages, burgers, caffeinated drinks, chips, chocolates, noodles, pizza, soft drinks, and sugar-sweetened drinks). Since junk food consumption increases the risk of altered metabolic profile [34–36], we decided to assess the junk food score during follow-up using the MEDSTYLE tool [32]. Briefly, the final score (junk score) is composed by 7 items (chips, fried potatoes, sweet snacks, bakery products, salty snacks, soft drinks, fruit juice) × frequency (never 0, rarely 1, once a week 2, 2–3 times a week 3, 4–5 times a week 4, daily 5) × portion (small 1, medium 2, large 3), ranging from 0 (no consumption) to 105 (the highest possible consumption).
- Assessment of the 21-item Depression Anxiety and Stress Scale questionnaire (DASS-21), which consists of three parts of 7 specific questions related to three related negative emotional states (depression, anxiety, and tension/stress) [37].

### Compliance with Ethical Standards

The authors declare the absence of conflict of interest. The research has been conducted in accordance with the Declaration of Helsinki, following approval of the local Ethics committee, University of Bari ‘Aldo Moro’ (study number 6752 ref. 1563 CE). The study was registered at ClinicalTrial.gov, and the Clinical Trial Registration Number NCT05430542. All patients gave full written informed consent. All authors had access to study data.

### Statistical analysis

The number of enrolled subjects was defined according to power calculation. For a comparison of two independent groups, 50 was the minimal sample size needed in each group to obtain a power of 0.80, when the effect size is medium and a significance level of 0.05 is employed. Following analysis of data distribution, continuous variables were expressed as median and range. Frequency and percentages were used for categorical variables. The chi-square test (proportions), the Mann–Whitney *U* test, the Wilcoxon signed-rank test, and the Kruskal–Wallis multiple comparison *Z* value test were employed, as appropriate, to evaluate intra‐or inter-group differences. All statistical analyses were performed using NCSS software (2021), and statistical significance was declared if a two-sided *P* value was < 0.05. Graphs were constructed with the SigmaPlot v. 14.05 version (Systat Software, Inc). Reporting of the study conforms to broad EQUATOR guidelines. The datasets generated and analysed during the current study are available from the corresponding author on reasonable request.

## Results

### Socio-demographic information

Enrolled subjects included 118 males and 127 females. These two groups were homogeneous for age distribution (males 44 years [range 20–58], females 40 years [range 20–58], *P* = 0.26).

According to the marital status, 55 subjects were single (*M*:*F* = 26:29, *P* = 0.88), 41 subjects living with family of origin (*M*:*F* = 25:16, *P* = 0.06), 21 married without children (*M*:*F* = 7:14, *P* = 0.15), 128 married with children (*M*:*F* = 60:68, *P* = 0.67) (Table 1S).

According to the occupational status, 63 subjects (26%) were “homeworkers” (only females), 126 subjects (51%) were involved in “remote work”, and 56 subjects (23%) in “essential activities” (health care, public safety, food and medicine stores), with a significant greater prevalence of males than females in both groups (*P* = 0.002, *P* < 0.001, respectively) (Table 1S).

### Lifestyle habits (Table 2S)

According to smoking habit, 137 subjects (56%) were not smokers, while 108 (44%) were smokers with no difference according to sex (*P* = 0.8).

According to alcohol consumption, 52 subjects (21%) used to consume alcoholic drink with a significant greater prevalence of males than females (*P* = 0.01), and the number of drink/day was greater in males than in females subjects (*P* = 0.015).

According to physical activity, 177 subjects (72%) were sedentary, while 68 subjects (28%) reported moderate physical activity with a significant greater prevalence of males than females in both groups (*P* = 0.0002).

### Anthropometric data at baseline

According to BMI, 182 subjects were overweight (*M*:*F* = 101:81, *P* = 0.0001; *M* 28.4 (range 25.7–29.9) vs. *F* 28.1 (range 25.1–29.8) Kg/m^2^, *P* = 0.18)), and 63 were obese (*M*:*F* = 17:46, *P* = 0.0001; *M* 30.5 (range 30–31.2) vs. *F* 30.8 (range 30–32.8) Kg/m^2^, *P* = 0.02)).

Waist circumference was significantly greater than the IDF cut-off both in male subjects (103 range (93–110) and in female subjects (91 (range 84–100) cm) (*P* < 0.0001).

### Comorbidities

Comorbidities and bio-humoral data of subjects are reported in Table 1. Fifty-five subjects (*M*:*F* = 33:22, *P* = 0.046) reported a diagnosis of type 2 diabetes, which was treated with oral anti-diabetic drugs. Among them, 23 subjects had a glycated hemoglobin concentration higher than the normal, with a significant difference between the two sexes (*P* = 0.007).

**Table 1:** Bio-humoral data of subjects (M/F) before and after COVID-19 lockdown

	Baseline			After COVID-19 lockdown		
	*M*	*F*	*P*	*M*	*F*	*P*
Glycemia (mg/dl)	106 (83–138)	108 (83–140)	0.91	102 (82–144)	109 (88–144)	0.01
Insulinemia (microUI/ml)	18 (11–27)	16 (10–28)	0.46	16 (10–32)	23 (10–33)	< 0.0001
Cholesterol (mg/dl)	174 (134–240)	179 (136–241)	0.81	180 (130–256)	186 (130–263)	0.08
LDL (mg/dl)	110 (72–166)	111 (70–168)	0.51	109 (72–167)	116 (66–173)	0.33
HDL Cholesterol (mg/dl)	40 (32–51)	42 (32–52)	0.002	40 (32–51)	41.3 ± 4.6	0.33
Triglycerides (mg/dl)	125 (55–265)	129 (45–320)	0.87	130 (40–305)	170 (55–360)	0.03
ALT (U/L)	43.5 (23–89)	45 (23–96)	0.45	44 (25–112)	52 (25–114)	0.029
AST (U/L)	38 (20–72)	38 (18–78)	0.81	38 (18–88)	44 (20–98)	0.04
GGT (U/L)	32 (20–68)	30 (20–73)	0.01	32 (22–124)	32 (20–108)	0.44

*P* Difference between Males and Females (Unpaired *T* test)

Eleven subjects (3 with diabetes mellitus) displayed a greater value of insulinemia than normal, with no difference between the two sexes (*P* = 0.4).

According to lipid profile, total serum cholesterol was abnormal in 71 subjects with no difference between the two sexes (*P* = 0.46). Among enrolled subjects, 35 were statin users for primary cardiovascular prevention. By following the criteria of metabolic syndrome, HDL cholesterol was low in 108 female subjects and in 61 male subjects. Triglycerides levels were increased in 90 subjects with no difference between two sexes (*P* = 0.87).

Serum ALT levels were higher than normal in 92 subjects, and comparable between the two sexes (*P* = 0.45). AST levels were normal and comparable between the two sexes (*P* = 0.81). GGT levels were normal and higher in males than in females (*P* = 0.01). According to the ultra-sonographic grading of fatty liver, 25 subjects showed normal liver at ultrasound, whereas 82 subjects showed grade I (mild), 109 subjects showed grade II (moderate), and 29 subjects showed grade III (severe) fatty liver (Table 2).

**Table 2:** Grading of liver steatosis of subjects (M/F) before and after COVID-19 lockdown

	Baseline			After COVID-19 lockdown			P*	*P°*	*P#*
	*M*	*F*	*P*	*M*	*F*	*P*			
Normal liver at ultrasound (N. of subjects)	8	17	0.08	6	9	0.5	0.1	0.6	0.1
Grade I (N. of subjects)	46	36	0.08	34	19	0.008	0.003	0.1	0.01
Grade II (N. of subjects)	52	57	0.2	45	38	0.2	0.02	0.2	0.01
Grade III (N. of subjects)	12	17	0.4	33	61	0.001	< 0.0001	0.0005	< 0.0001

*P* Difference between Males and Females (Unpaired *T* test)

P* Difference between baseline and after lockdown in all subjects (paired *T* test)

*P°* Difference between males at baseline and after lockdown (paired *T* test)

*P#* Difference between females at baseline and after lockdown (paired *T* test)

### Adherence to MD

At the baseline, the adherence to MD was significantly greater in males than in females (*M* 14, range 9–17) vs *F* 13, range 10–18), *P* < 0.0001).

## Effects of the COVID-19 lockdown

### Lifestyle habits

Following the lockdown period, no changes were detected according to smoking habit (i.e., number of smokers) in both sexes. The number of alcohol drinkers increased in both sexes (52 subjects vs. 135, *P* < 0.001; *M*:*F* = 61:74), with no sex difference (*P* = 0.3). Moreover, no changes were detected for physical activities.

### Anthropometric data

**Fig. 2:**
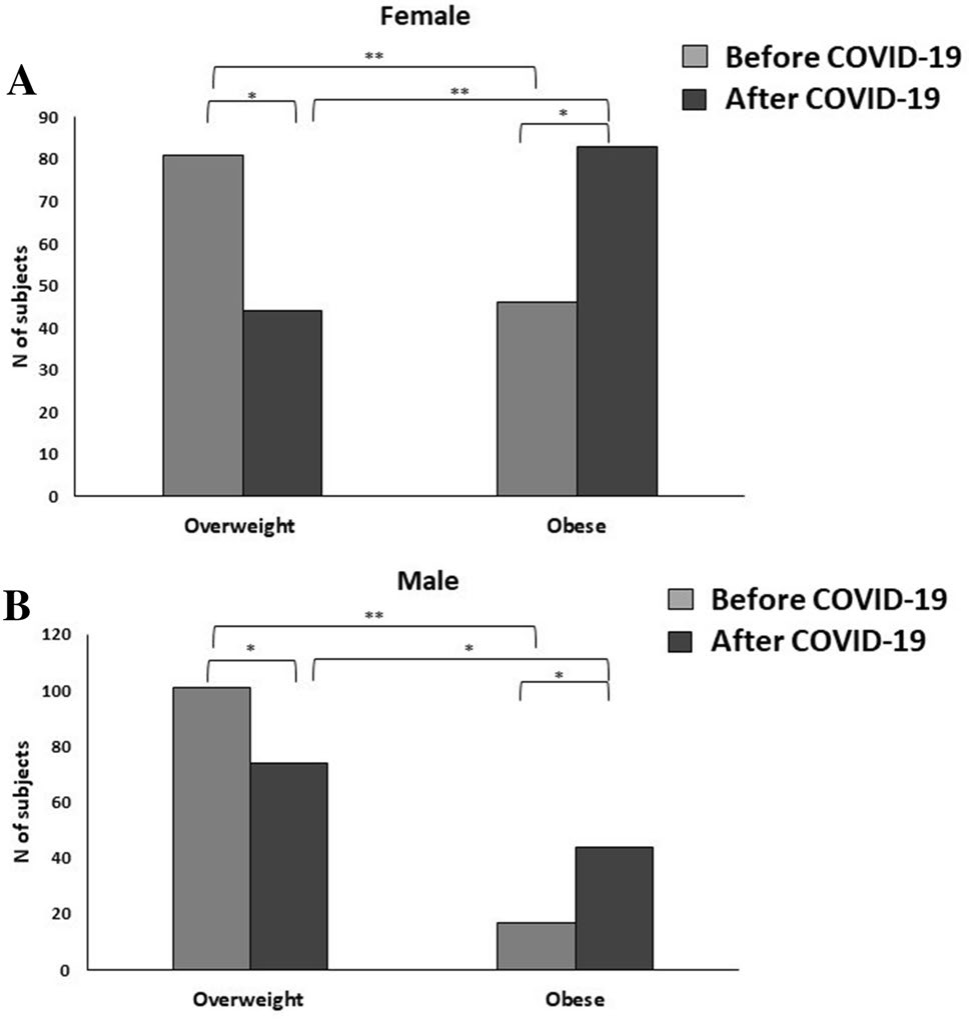
Number of female (**A**) and male (**B**) subjects classified in overweight and obese groups before and after COVID-19 lockdown. *P < 0.05; **P > 0.01: Chi‐square test (proportions)

After the COVID-19 lockdown, the number of overweight subjects dramatically decreased to 117 (*M*:*F* = 74:43, *P* < 0.0001; BMI values *M* 28.4 (range 25.6–29.8) vs. *F* 28.2 (range 25.1–29.8) Kg/m^2^, *P* = 0.5). By contrast, the number of obese subjects significantly increased in both sexes, and was more pronounced in females than in males both according the percentage of obese subjects and value of BMI (*M*:*F* = 44:83, *P* < 0.0001; *M* 30.9 (range 29.9.1–33.1) vs *F* 32.5 (range 30.1–35.8) Kg/m^2^, *P* < 0.0001) (Fig. 2).

Waist circumference increased in both sexes with respect to baseline (*M* 105 (range 93–115) vs *F* 95 (range 84–103) cm, *P* < 0.0001).

### *Comorbidities (Table **1**)*

After lockdown the number of diabetic subjects with elevated value of glycated hemoglobin increased from 23 subjects to 42 (*P* = 0.01) (*M*:*F* = 25:17, *P* = 0.1). After lockdown there was no difference in the percentage of males with hyperinsulinemia (2% vs. 5%, *P* = 0.21), while the percentage of females significantly increased (45% vs. 5%, *P* < 0.0001).

HDL cholesterol levels lower than the cut-off was detected in a higher percentage in females, when compared to baseline (78% vs. 97%, *P* < 0.0001), with no significant difference according to the value (*P* = 0.22), while any difference in male subjects was detected according to the percentage of subjects and value of HDL (*P* = 0.55). Triglycerides levels increased after the lockdown in male and female subjects, showing significant higher values in females than in males (*P* = 0.03).

According to liver function, ALT levels significantly increased in both sexes (*M*, *P* = 0.001; *F*, *P* < 0.0001), and was significant higher in females than in males (*P* = 0.029), while there was no difference in percentage of affected individuals intra-sexes (*M* 85% vs. 86%, *F* 72% vs. 79%, *P* = 0.32).

AST levels were higher than normal value in 4 females (98 (range 90–102)) and in one male subject (88) U/L.

GGT levels were increased in 3 male and in 2 female subjects (*M* 113 range (*M*: 102–124), *F* 98.5 (range 89–108)) U/L.

The grading of liver steatosis is reported in Table 2. Fifteen subjects (*M*:*F* = 6:9, *P* = 0.5) had normal liver at ultrasound at the ultrasound examination, 53 subjects showed grade I (mild), 83 subjects showed grade II (moderate), and 94 subjects showed grade III (severe). The number of subjects with normal liver at ultrasound, mild steatosis, and moderate steatosis decreased after lockdown compared to baseline, while the number of subjects with severe steatosis remarkably increased after lockdown (from 29 to 94 subjects, *P* < 0.0001). The decrease in mild/moderate steatosis and the consecutive increase of severe steatosis were more pronounced in female group (Table 2).

### Adherence to MD

**Fig. 3:**
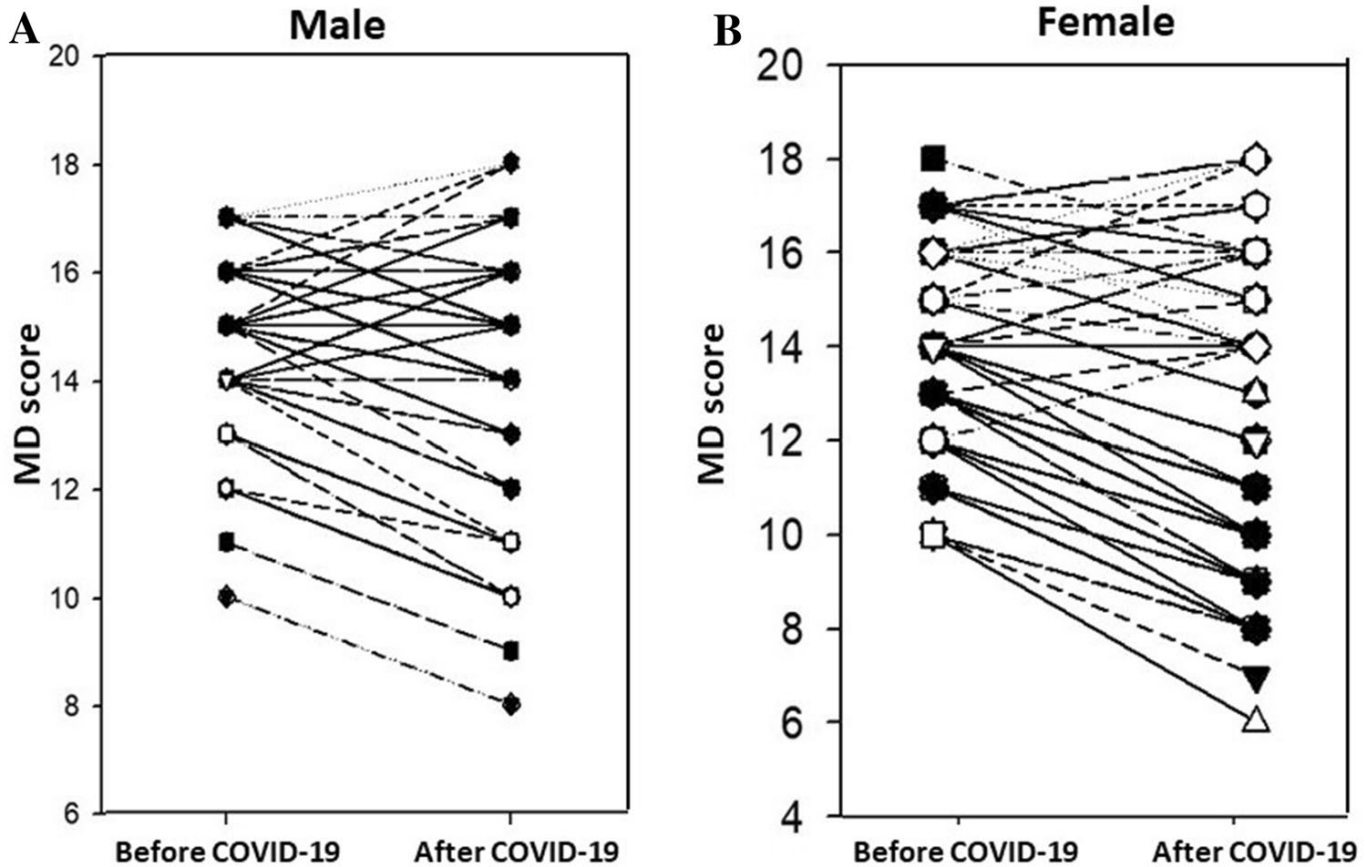
Variance in MD adherence score between male (**A**) and female (**B**) before and after COVID-19 lockdown

The MD score significantly decreased after lockdown in female subjects (from 13 (range 10–18) to 11 (range 6–18) points, *P* = 0.012) and slightly changed in males (*M* 14 (range 9–17) vs. 14 (range 8–18) points, *P* = 0.22). Moreover, the MD score after lockdown was significantly lower in females than in males (*P* < 0.0001) (Fig. 3).

### Junk food score

Junk food score was significantly greater in female than in male subjects (*M* 25 (range 0–85) vs *F* 65 (range 0–90) *P* < 0.0001) (Table 3).

**Table 3:** Psychological profile and Junk Food according to sex

	After COVID-19 lockdown		
	*M*	*F*	*P*
Depression	8 (5–16)	14 (7–29)	< 0.0001
Anxiety	11 (5–18)	16 (8–20)	< 0.0001
Stress	18 (10–29)	26 (15–33)	< 0.0001
Junk food	25 (0–85)	65 (0–90)	< 0.0001

*P* Difference between Males and Females (Unpaired *T* test)

### Psychological profile

The scores of depression, anxiety, and tension/stress were significantly greater in females than in males (*M* 8 (range 5–16) vs *F* 14 (range 7–29), *P* < 0.0001; *M* 11 (range 5–18) vs *F* 16 (range 8–20), *P* < 0.0001; *M* 18 (range 10–29) vs *F* 26 (range 15–33), *P* < 0.0001, respectively), by showing an extremely severe profile in both sexes (Table 3).

### Impact of work activities and social/familial status

Regarding work activities during COVID-19 lockdown, subjects were divided as “home work” (63 subjects), “remote work” (126 subjects), and “essential activities” (56 subjects). Therefore, BMI, MD adherence, junk score, and aspects of psychological profile were evaluated at baseline and after lockdown. The BMI for “home work” group (all females) significantly increased from 29.4 (range 25.6–32.8) at baseline to 31.9 (range 26.3–35.8) Kg/m^2^ (*P* < 0.0001) after lockdown. The BMI for “remote work” groups significantly increased in all subjects (28.7 (range 25.2–32.3) vs. 30.3 (range 24.5–34.6) Kg/m^2^ (*P* < 0.0001)), in the males group (28.7 (range 26.3–30.8) vs. 29.8 (range 25.7–33.3) Kg/m^2^ (*P* < 0.0001)), and in the females group (29 (range 25.2–32.4) vs. 31.5 (range 24.5–34.6) Kg/m^2^ (*P* < 0.0001)), with a greater increase of BMI (calculated as delta BMI) in females than in males (2.2 (range –0.77 to 4.9) vs. 1.6 (range –1.9 to 2.7) Kg/m^2^, *P* < 0.0001)).

The BMI for essential worker group significantly increased in all subjects (28.1 (range 25.1–31.2) vs. 28.4 (range 25.1–32.8) Kg/m^2^ (*P* = 0.009)). Also, in the males group BMI significantly increased (28.4 (range 25.7–31.2) vs. 29 (range 25.6–32.8) Kg/m^2^ (*P* = 0.0005)), while no difference was detected in the females group (26.3 (range 25.1–30.4) vs. 27.3 (range 25.1–29.4) Kg/m^2^ (*P* = 0.75)), as well as according to delta BMI between males and females (0.32 (range –0.7 to 2.9) vs. 0.34 (range –1.2 to 1.5) Kg/m^2^, *P* = 0.23)).

Moreover, changes in MD adherence score were also observed in different work sub-groups before and after lockdown. In details, the MD score for “home work” group significantly decreased from 13 (range 10–17) at baseline to 10 (range 7–18) (*P* < 0.0001) after lockdown.

MD adherence score for “remote work” groups significantly decreased in all subjects (14 (range 10–18) vs. 11 (range 6–18) (*P* < 0.0001)), in the males group (14 (range 10–17) vs. 13 (range 8–18) (*P* < 0.0001)), in the females group (13 (range 10–18) vs. 10 (range 6–18) (*P* < 0.0001)), with a greater decrease (calculated as delta MD adherence score) in females than in males (1 (range –8 to 7) vs. 3 (range –5 to 7), *P* < 0.0001)).

The MD adherence score for “essential worker” group significantly decreased in all subjects (15 (range 9–17) vs. 15 (range 8–18) (*P* = 0.0004)). Also, in the males group MD adherence score significantly decreased (15 (range 9–17) vs. 14 (range 8–18) (*P* = 0.0002)), while no difference was detected in the females group (15 (range 12–17) vs. 16 (range 12–18) (*P* = 0.27)), as well as according to delta MD adherence score between males and females (0 (range –6 to 5) vs. –3 (range –4 to 2), *P* = 0.2)).

Junk food score for “home work” group (only females) was 70 (range 0–90). Junk food score for “remote work” group was 55 (range 0–85), and significantly greater in the females (65 range 0–85) than in males (45 range 0–85) (*P* < 0.0001), while Junk food score for “essential worker” group was 0 (range 0–60), and comparable between the two groups (*M* 0 range 0 – vs. *F* 0 range 0–35) (*P* = 0.8). Overall, Junk food score significantly decreased from “home work” to “remote work” to “essential work” (*P* < 0.0001).

The scores of depression, anxiety, and tension/stress for “home work” group (only females) were 14 (7–29), 18 (8–20), and 27 (15–32), respectively.

The scores of depression, anxiety, and tension/stress for “essential worker” group were 9 (range 6–21), 12 (range 5–20), and 18 (10–33), respectively. Moreover, all the scores were significantly greater in the female group (13 range 7–21; 16 range 9–20; 27 range 15–33) than in males (8 range 6–16; 10 range 5–18; 17 range 10–25) (*P* < 0.0001), respectively. The three scores for “remote work” group were 8 (range 5–14), 13 (range 7–18), and 24 (range 13–29), respectively. The scores of depression, and anxiety for “remote work” group were significantly greater in the females group (10 range 8–14; 14 range 13–17) than in males (8 range 5–12; 12 range 7–18) (*P* = 0.0002; *P* = 0.006, respectively), while no significant difference was detected according to the score of tension/stress (*F* 25 range 19–26 vs. *M* 22 range 13–29) (*P* = 0.1). Overall, depression score decreased significantly from “home work” to “remote work” to “essential work” (*P* < 0.0001), while the anxiety and tension/stress scores were significantly higher (*P* < 0.0001) in “home work” group than in other two groups without any significant change between “remote group” and “essential group”.

Then, participants were sub-divided by subjects with children (129 subjects) and without children (116 subjects). The BMI of subjects without children was 28.5 (range 25.1–32.8), and comparable between males and females (*M* 28.4 (range 25.7–31.2) vs. *F* 28.6 (range 25.1–32.8) Kg/m^2^) (*P* = 0.5) at baseline, increased significantly after lockdown (29.1 (range 24.5–35.1) Kg/m^2^ (*P* < 0.0001), and was significantly greater in females than in males (*F* 29.4 (range 24.5–35.1) vs. *M* 28.4 (range 25.6–32.2) Kg/m^2^)) (*P* = 0.02).

The BMI of subjects with children was 29.1 (range 25.5–32.5), and was significantly higher in females than in males (*F* 29.4 (range 25.5–32.5) vs. *M* 29 (range 26.5–30.8) Kg/m^2^) (*P* = 0.01) at baseline, increased significantly after lockdown (31.2 (range 25.4–35.8 kg/m^2^) (*P* < 0.0001), and was significantly greater in females than in males (*F* 31.9 (range 25.4–35.8) vs. *M* 30.3 (range 26.9–33.3) Kg/m^2^) (*P* < 0.0001). The increase in BMI (delta BMI) was significantly greater in the subjects with children than in those without children (2.2 (range –1 to 4.9) vs. 0.7 (range –1.9 to 5.4) Kg/m^2^, *P* < 0.001).

At baseline, the MD score of subjects without children was higher in males than females (*P* = 0.005), after lockdown decreased significantly (*P* < 0.0001), and the MD score was significantly greater in males than females (*P* = 0.01) (Table 3S).

At baseline, the MD score of subjects with children was significantly higher in males than in females (*P* = 0.0005). After lockdown the MD score decreased significantly (*P* < 0.0001) and was significantly greater in males than in females (*P* < 0.0001). The decrease in MD score (delta MD) was significantly greater in the subjects with children than in those without children (*P* < 0.0001) (Table 3S).

Junk food score for subject was significantly greater in females than in males both in the groups without children (*P* < 0.0001), and with children (*P* < 0.0001), Overall, Junk food score increased significantly from subjects without children to subject with children (*P* < 0.0001) (Table 3S).

According to the psychological profile, all the scores were significantly greater in the female group than in males (*P* < 0.0001), respectively. Overall, depression, anxiety, and tension/stress scores were significantly higher (*P* = 0.008; *P* = 0.001, *P* < 0.0001, respectively) in subjects without children than subjects with children, and the changes in psychological scores were more remarkable in females than males (Table 3S).

## Discussion

In the present study, we had the opportunity to evaluate the impact of COVID-19 lockdown on metabolic and psychological profile in a cohort of overweight/obese adult subjects in the presence of one or more of dysmetabolic features (i.e., diabetes, hypertension, metabolic-associated liver steatosis, increased waist circumference, and dyslipidemia). The general profile showed that a half of subjects were married with children, the large part was involved in remote work, and a quarter in essential activities.

Regarding to lifestyle habits, at baseline, only 21% used to consume alcohol with a greater prevalence of males than females, while after lockdown the percentage of consumers increased in both sexes. The impact of COVID-19 social restrictions on the amount of alcohol consumption was shown by several studies [38–40]. The use of alcohol is linked to its anxiolytic, antidepressant, relaxing and sedative role, but exerts dangerous physical and psychological effects [41]. The stress during isolation as well as the social crisis affects behavioral patterns, and represents strong risk factors for the onset and spike of alcohol misuse [40, 42]. Our population showed weight gain and worsening of metabolic patterns (i.e., glycemic and lipid profile) during the lockdown, which were associated with bad lifestyle and greater in females than in males. Our findings are in line with previous studies, which reported how the unhealthy dietary habits during the lockdown had negative impact on body weight [43–46].

The reported unhealthy dietary habits were characterized by consumption of junk foods and lower adherence to the MD, mostly in females that were homeworkers or involved in remote work, and with children. These results are consistent with the worldwide picture. As shown by previous studies, several factors played a role in changes of dietary habits, and included socio-economic resources, availability and marketing of foods, lower access to healthcare, taking care of family members, psychological disturbances [47, 48].

In the present study, the score of depression, anxiety and stress showed a severe psychological impairment of our population, which was more pronounced in females than in males. Moreover, values by DASS-21 were at least twice those obtained from previous studies [49–51]. This discrepancy could be because most of the studies which used DASS-21 were conducted at the beginning of the lockdown, and not after a prolonged period of isolation. By contrast, our results are in line with those from other studies, which showed an increasing level of psychological scores with the prolongation of the period of isolation [52, 53]. Moreover, our cohort is known to be more at risk for anxiety and depression than the general population [52]. The greater psychological distress of females than males in our population is in line with other studies [54–57] reflects a higher psychological vulnerability to traumatic and adverse conditions, as well as the strong commitment of females to family activities [55].

The high level of stress in response to the COVID-19 pandemic was associated with unhealthy eating strategies to cope [58], characterized by consumption of comfort foods (i.e., high fat, energy dense and palatable snack foods) [59–61].

Although evidence suggests that COVID-19 lockdown has influenced the emotional status, we have to consider with caution this aspect also in our study because of the lack of data on the mental status before the lockdown, and the need for longer follow-up.

Moreover, our population showed a sedentary profile, which did not change before and during the lockdown. The main reasons are the low level of physical activity at baseline which is typical of overweight/obese subjects, [48], and the negative impact of the social isolation, the large increase in sitting time, and stress on exercise [62].

Although the impact of COVID-19 pandemic on work and family is in no doubt, here we evaluated the impact of work and family status on the change of weight gain (evaluated as BMI), dietary habit (adherence to MD) and psychological aspects. In this regard, subjects within “home work” group, mostly housewife subjects, with respect to other groups, reported a higher increase of BMI, decrease on MD adherence, increase of junk food consumption, and worsen psychological profile. These findings are in accordance with previous studies that showed higher mental health problems in adult people with home work [24, 63–66]. In the other hand, the same trend was observed in subjects with children compared to subjects without. Our findings have revealed that for many “child-care” parents, especially housewife subjects experienced an increase of body mass index due to the decrease of physical activities and increase junk food consumption with unhealthy dietary habit.

## Limitations

This study has some limitations. The first limitation of the present study was that all subjects were enrolled in a single clinical center, and in a limited geographical area. This aspect can potentially limit the generalizability of results. Further studies are therefore needed to expand findings deriving from the present study in larger and more heterogeneous geographical and socio-economic contexts. Moreover, the results according to alcohol consumption, physical activity, dietary habits, and levels of psychological profile are based on self-reported data, which may be affected by information bias.

## Conclusions

The COVID-19 lockdown represented a public health trauma becoming a strong challenge to psychological resilience. The present study suggests that the weight gain and worsened metabolic profile of overweight/obese subjects were the consequence of the increased consumption of unhealthy dietary patterns and the sedentary lifestyle. We can speculate that the adopted lifestyle during the lockdown could represent the individual strategy toward high levels of depression, anxiety, and stress.

Gender had a distinct outcome on metabolic and health status. Females, especially housewives and those with children, had the worst metabolic and psychological profile. This aspect likely depended on low adherence to the Mediterranean diet and to high junk food consumption.

We learned from the COVID-19 lockdown that it is necessary to promote preventive policies focusing on gender differences to keep the healthy status. In addition, even during periods of home confinement, these preventive policies must guarantee appropriate health care services in subjects with high risk of cardio-metabolic disorders.

## Supplementary Information

Below is the link to the electronic supplementary material.

Figure 1S: Flow-chart of the study (see text for details). Supplementary file1 (JPG 90 KB)

Supplementary file2 (DOCX 16 KB)

## Data Availability

Data are available on motivated request due to privacy or other restrictions. The data that support the findings of this study are available on request from the corresponding author [Prof Leonilde Bonfrate]. The data are not publicly available due to human data sensibility and privacy (data containing information that could compromise research participant privacy/consent).

## Abbreviations

BMI: Body mass index
DASS-21: Depression Anxiety Stress Scale-21
MD: Mediterranean diet
SARS-CoV-2: Severe acute respiratory syndrome coronavirus 2
WHO: World Health Organization
