# Supplementary figures and images for: Gender-dependent impact of COVID-19 lockdown on metabolic and psychological aspects
Source: Intern Emerg Med. 2023 Jan 27;18(2):385–95. doi: 10.1007/s11739-022-03173-9 (PMC9879743; doi:10.1007/s11739-022-03173-9)

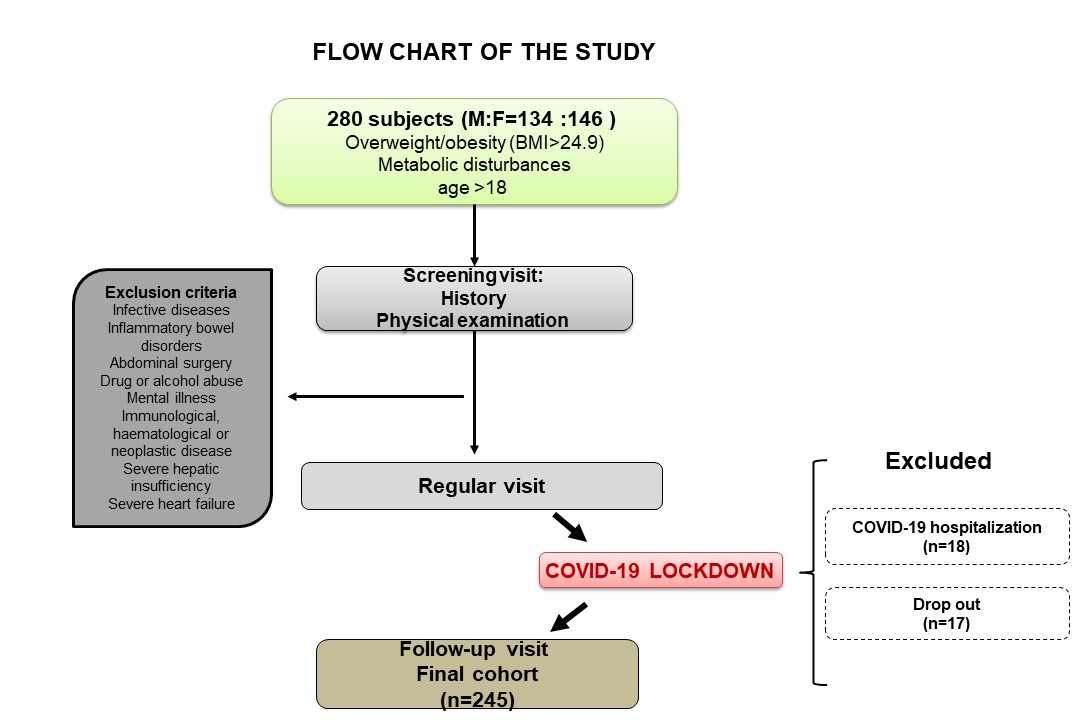

Supplement: Supplementary file 1 — Figure 1S: Flow-chart of the study (see text for details). Supplementary file1 (JPG 90 KB) [file 11739_2022_3173_MOESM1_ESM.jpg]
