# Supplementary material for: Gender-dependent impact of COVID-19 lockdown on metabolic and psychological aspects
Source: Intern Emerg Med. 2023 Jan 27;18(2):385–95. doi: 10.1007/s11739-022-03173-9 (PMC9879743; doi:10.1007/s11739-022-03173-9)
Supplement: Supplementary file 2 — Supplementary file2 (DOCX 16 KB) [file 11739_2022_3173_MOESM2_ESM.docx]

Table 1S: Stratification of the subjects according to the marital status, and occupational status at baseline

|  |  | Baseline | | |
| --- | --- | --- | --- | --- |
|  |  | *M* | *F* | *P* |
| Single | N. of subjects | 26 | 29 | 0.88 |
|  | Age (yrs) | 39.5  (range 31-45) | 33  (range 31-40) | 0.003 |
| living with family of origin | N. of subjects | 25 | 16 | 0.06 |
|  | Age (yrs) | 24  (range 20-29) | 27  (range 20-29) | 0.4 |
| married without children | N. of subjects | 7 | 14 | 0.15 |
|  | Age (yrs) | 38  (range 31-44) | 40.5  (range 38-48) | 0.09 |
| married with children | N. of subjects | 60 | 68 | 0.67 |
|  | Age (yrs) | 52  (range 36-58) | 45  (range 33-58) | 0.0001 |
| homeworkers | N. of subjects | - | 63 | - |
|  | Age (yrs) | - | 44 yrs  (range 21-58) | - |
| remote work | N. of subjects | 73 | 53 | 0.002 |
|  | Age (yrs) | 44  (range 20-58) | 39  (range 29-56) | 0.03 |
| essential activities | N. of subjects | 45 | 11 | <0.001 |
|  | Age (yrs) | 44  (range 20-57) | 36  (range 20-45) | 0.15 |

*P:* Difference between Males and Females (unpaired T-test)

Table 2S: Lifestyle habits at baseline

|  |  | Baseline | | |
| --- | --- | --- | --- | --- |
|  |  | *M* | *F* | *P* |
| Smoking habit (N°) | No Smokers | 65 | 72 | 0.8 |
|  | Smokers | 53 | 55 | 0.8 |
| Alcohol consumption | N. of subjects | 33 | 19 | 0.01 |
|  | Number of drink/day | - 0 (range 0-3) | - 0 (0-2) | 0.015 |
| Physical activity | Sedentary  (N. of subjects) | 72 | 105 | 0.0002 |
|  | Moderate physical activity  (N. of subjects) | 46 | 22 | 0.0002 |

*P:* Difference between Males and Females (chi-square test)
